# Balloon-assisted crossing via *trans*-radial access during transcatheter aortic valve implantation: a case series demonstrating the BACTRA technique

**DOI:** 10.1093/ehjcr/ytag536

**Published:** 2026-08-11

**Authors:** Garry W Hamilton, Mehdi Eskandari, Alexandros Papachristidis, Jonathan Byrne, Philip MacCarthy, Rafal Dworakowski

**Affiliations:** Department of Cardiology, King’s College Hospital, Denmark Hill, London SE5 9RS, UK; Department of Cardiology, King’s College Hospital, Denmark Hill, London SE5 9RS, UK; Department of Cardiology, King’s College Hospital, Denmark Hill, London SE5 9RS, UK; Department of Cardiology, King’s College Hospital, Denmark Hill, London SE5 9RS, UK; Department of Cardiology, King’s College Hospital, Denmark Hill, London SE5 9RS, UK; Department of Cardiology, King’s College Hospital, Denmark Hill, London SE5 9RS, UK; Cardiology Department, Medical University of Gdansk, Dębinki 7, Gdańsk 80-211, Poland

**Keywords:** Case report, TAVI, TAVR, Transradial, Balloon crossing, Vascular access

## Abstract

**Background:**

Crossing the aortic valve with the device during transcatheter aortic valve implantation (TAVI) can be challenging, with several troubleshooting techniques described. Balloon-assisted valve crossing is commonly considered, although the need for a second femoral access of sufficient size to accommodate the valvuloplasty balloon limits its appeal.

**Case summary:**

In this two-case series, we demonstrate the feasibility of balloon-assisted aortic valve crossing for both balloon-expandable and self-expanding TAVI platforms using percutaneous transluminal peripheral angioplasty balloons delivered via *trans*-radial access. Where difficulty was encountered with crossing the valve and basic techniques were unsuccessful, the BACTRA (Balloon-Assisted Crossing via Trans-Radial Access) technique facilitated valve crossing and successful valve deployment. In Case 1, the inability to cross the valve related predominantly to severe tortuosity throughout the arterial tree and a subsequent lack of torque and device manipulation to negotiate the native valve. In Case 2, the issue largely related to the horizontal aorta. Both patients had successful valve deployment and were subsequently discharged well.

**Discussion:**

Regardless of the underlying cause or the TAVI platform utilized, this report demonstrates that the BACTRA technique has several plausible benefits. Valve crossing was facilitated in both cases without additional arterial punctures or sheath upsizing, allowing the procedures to proceed efficiently and safely without any added risk of vascular complications. Procedural steps, troubleshooting, and equipment compatibility are outlined in this case series.

Learning points

- Peripheral percutaneous transluminal angioplasty (PTA) balloons delivered via *trans*-radial access (TRA) can facilitate balloon-assisted aortic valve crossing.
- The BACTRA (Balloon-Assisted Crossing via Trans-Radial Access) technique should be considered in the difficult crossing algorithm where TRA is used as secondary access.
- Following standard delivery system manipulation techniques and use of a buddy wire, BACTRA can then be attempted over the buddy wire already in the left ventricle.
- BACTRA avoids additional arterial access, likely lowering vascular complication rates, and avoids loss of wire position (for nosecone snaring) or the need for complete device removal.

## Introduction

Transcatheter aortic valve implantation (TAVI) is now the standard of care for many patients with significant aortic stenosis, particularly those at high surgical risk.^1^ Crossing the native valve with the delivery system can pose challenges, even where balloon aortic valvuloplasty (BAV) has already been performed. Case duration, complexity, and procedural risk consequently increase given that the additional equipment required can be associated with complications.

Several techniques to facilitate difficult aortic valve crossing have been described including standalone or optimized BAV, buddy wires, balloon-assisted crossing, and snaring of the delivery system nosecone.^2–4^ Each has limitations and risks and usually requires an additional arterial sheath of sufficient size to deliver equipment. Usually facilitated via the contralateral common femoral artery, the patient is then exposed to an increased risk of bleeding and vascular complications that would ideally be avoided.

Rather than using a valvuloplasty balloon approximated to the annular dimension, successful balloon-assisted aortic valve crossing usually needs only minimal displacement of the device nosecone. Further, following the rise and now routine use of *trans*-radial access (TRA) for percutaneous coronary intervention,^5,6^  *Trans*-radial access is increasingly adopted for secondary access in TAVI. At our institution, TRA is now used routinely instead of contralateral femoral access. This case series demonstrates that small transluminal angioplasty balloons (PTA) designed for peripheral interventions delivered via TRA can effectively facilitate aortic valve crossing. Two cases are presented, one of a balloon-expandable (BE) and the other a self-expanding (SE) TAVI platform. The need for additional arterial access was eliminated, and both cases proceeded efficiently without complications. We outline the required equipment, sheath compatibility of various PTA balloons, and procedural steps to perform the BACTRA (Balloon-Assisted Crossing via Trans-Radial Access) technique.

## Summary figure

**Figure:**
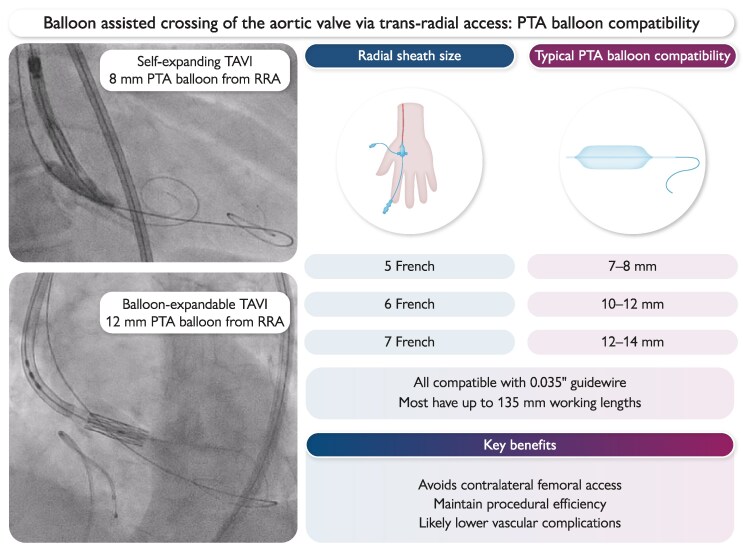
Balloon-assisted crossing of the aortic valve via *trans*-radial access: PTA balloon compatibility.

## Case 1

**Figure 1:**
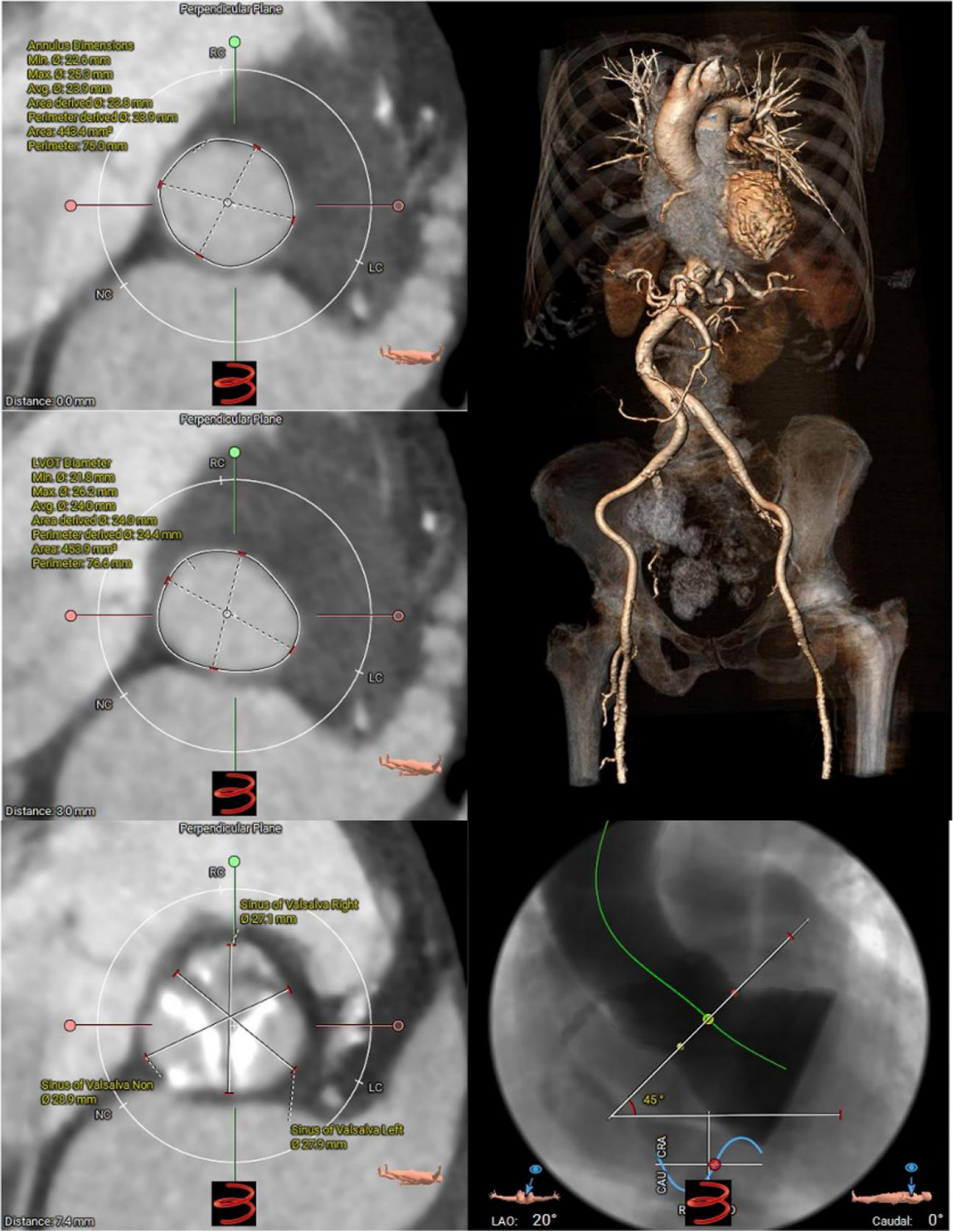
Case 1: CT TAVI for Case 1. Case planning highlighted significant tortuosity of the iliac vessels and aorta. A 23 mm Sapien was chosen based on the sizing in the upper end of the range, very severe leaflet calcification, and no LVOT or annular calcification.

A 94-year-old independent lady was admitted with heart failure on a background of months of exertional dyspnoea and lethargy. Transthoracic echocardiography showed new left ventricular dysfunction with an ejection fraction of 27% and severe aortic stenosis. The aortic valve was tricuspid with a peak/mean gradient of 96/65 mmHg despite low flow. The calculated valve area was 0.6 cm^2^. A CT TAVI confirmed a heavily calcified tricuspid valve with a calcium score of 2577 AU and no annular or left ventricular outflow tract (LVOT) calcification (*Figure 1*). Femoral access was feasible, although significant vascular tortuosity and extensive aortic calcification were noted. Given her symptoms, a heart failure hospitalization, and new severe LV dysfunction, inpatient TAVI was planned. A 23 mm Sapien Resilia valve (Edwards Lifesciences) was chosen with the aim of limiting the valve deployment time to a single rapid pacing run and to avoid any difficulty that may be encountered with positioning a SE valve in very tortuous anatomy.

### Procedure

Left transfemoral access was chosen based on the tortuosity, particularly the angulation of the infra-renal aorta and its relationship to the common iliac trajectories. Secondary access was via right TRA through which a pigtail catheter was placed in the aortic root. No pre-TAVI BAV was performed. Advancement of the valve and delivery system through the sheath into the infra-renal aorta was challenging (see Supplementary material online, *Video S1*). Supplementary material online, *Video S2* highlights the further tortuosity throughout the abdominal and thoracic aorta which made device manipulation difficult. Several attempts to cross the valve were unsuccessful including approaching from different trajectories, varied flex and rotation of the delivery system, and inflation of the deployment balloon 1–2 mL to create a smoother transition from nosecone to the crimped valve. A buddy wire with these strategies was also unsuccessful. The inability to cross related to angulation and severe vascular tortuosity limiting transmission of torque and thus manipulation of the nosecone. The delivery system was retracted back to the ascending aorta. Balloon-assisted aortic valve crossing of the transcatheter heart valve through the leaflets was facilitated via right TRA with a 12 × 40 mm Powerflex PTA balloon (Cordis) (see Supplementary material online, *Video S3*). The valve was then deployed in a standard fashion under rapid pacing. An on-table aortogram was satisfactory (see Supplementary material online, *Video S4*), and an echocardiogram showed a well-seated valve with no paravalvular leak. The peak/mean gradient was 7/4 mmHg.

## Case 2

**Figure 2:**
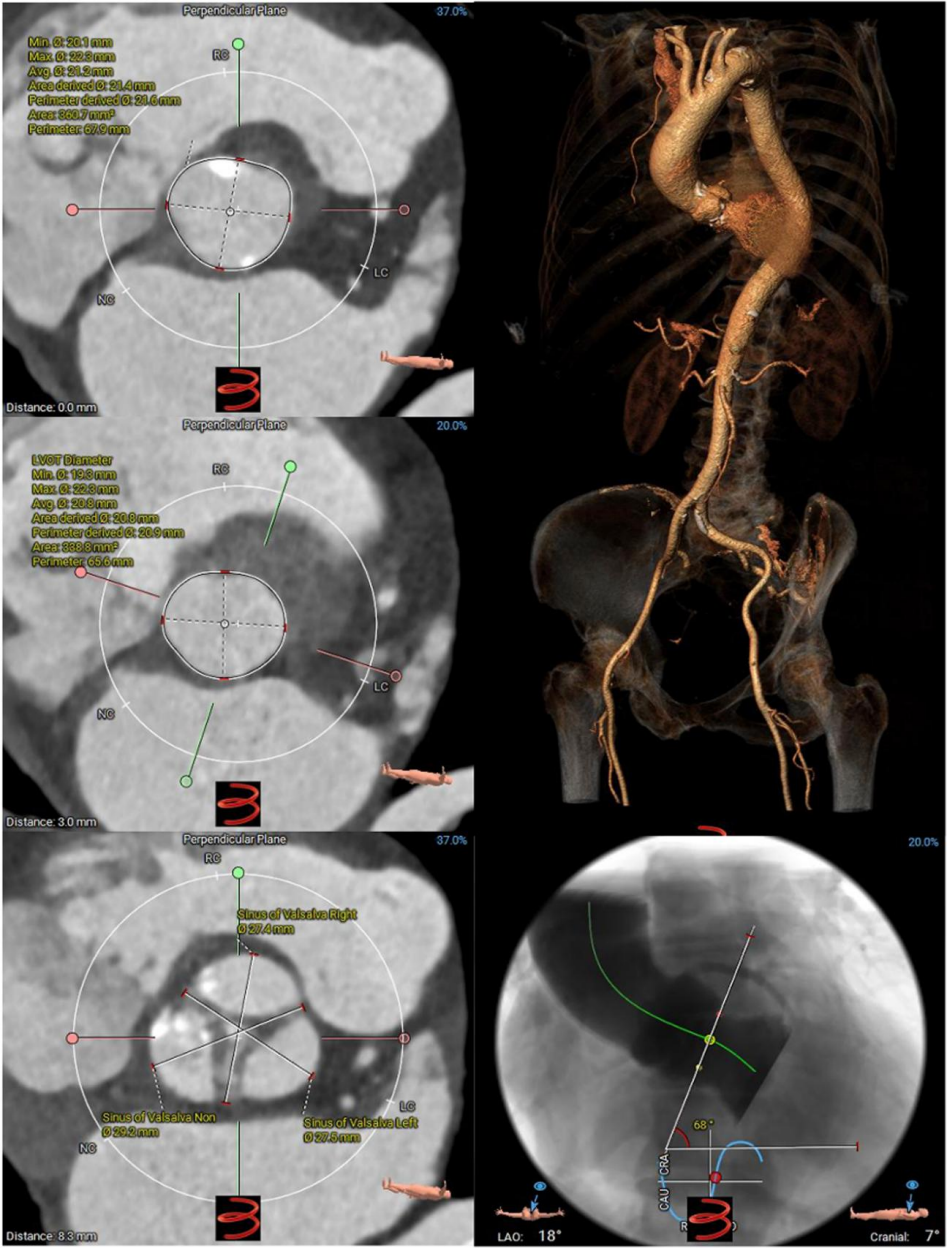
Case 2: CT TAVI for Case 2. Case planning identified the very horizontal aorta; however, a self-expanding system was preferred given the significant annular calcium.

An 86-year-old independent lady with no significant history presented for TAVI in the context of severe symptomatic aortic stenosis. Echocardiography showed mild left ventricular dysfunction (LV ejection fraction, 48%). The aortic valve was tricuspid with a peak/mean gradient of 88/56 mmHg and a calculated valve area of 0.5 cm^2^. A CT TAVI revealed a calcium score of 3944 AU. There was annular calcium with a short extension into the LVOT (*Figure 2*). Transfemoral access was feasible. Although it was noted that the aortic root and proximal ascending aorta were horizontal, a 25 mm Navitor (Abbott Medical) SE platform was chosen based on the perceived rupture risk with a BE valve related to the annular and LVOT calcification.

### Procedure

Via right TRA, a pigtail was placed in the non-coronary cusp, while the main access was via the right common femoral artery. The procedure progressed uneventfully including BAV with a 20 mm VACS II balloon (Osypka). The 25 mm Navitor was then advanced with the FlexNav delivery system into the aortic root; however, the native valve could not be crossed using the same algorithm described in Case 1. Balloon-assisted aortic valve crossing was then efficiently performed with an 8 × 40 mm Powerflex PTA balloon delivered from the 6-Fr right TRA sheath (see Supplementary material online, *Video S5*). The valve was then successfully deployed despite the horizontal aorta (see Supplementary material online, *Video S6*). Invasive haemodynamics and an on-table echocardiogram showed no paravalvular leak and satisfactory gradients.

Summary of procedural steps for the BACTRA technique to facilitate aortic valve crossing with the delivery system during TAVI:

1. The TAVI system is retracted into the upper ascending or descending aorta with the guidewire kept in the left ventricle.
2. The pigtail catheter via TRA is exchanged for an appropriate catheter to cross the valve. Once crossed, a 0.035′ exchange-length stiff wire with a pre-shaped curve is advanced into the LV.
3. The PTA balloon is advanced over the wire across the aortic valve, ideally against the outer curve. The size of the PTA balloon and therefore the need for a 6- or 7-Fr radial sheath are at the discretion of the operator based on the clinical picture and degree of displacement necessary.
4. Once across the valve traversing the leaflets and annular plane, the PTA balloon is inflated (rapid pacing usually not necessary). Ideally, the balloon has occupied the space where the guidewire and valve nosecone had been biasing and getting stuck. The delivery system is advanced across the valve (Summary figure; Supplementary material online, *Videos S3* and *S5*). The PTA balloon is deflated as the nosecone crosses into the LVOT and the device moves into the annular plane. Where crossing is not facilitated, earlier deflation may facilitate delivery system crossing if gentle forward traction is applied as the PTA balloon deflates.
5. If difficulty is encountered, the most common issue relates to the position of the balloon relative to the guidewire and nosecone, whereby the PTA balloon is not on the outer curve. Coordinated traction on the wire and balloon catheter with a combination of balloon inflation or deflation may all assist in facilitating optimal positioning and subsequent valve crossing.
6. Once the valve has been crossed, the PTA balloon and guidewire are removed from the left ventricle to ensure this wire is not jailed when the valve is deployed. The pigtail can then be re-positioned in the aortic root to proceed with the case in a standard fashion.

## Discussion

Vascular tortuosity, aortic root angulation, pattern of calcification, and valve morphology can all suggest that difficulty may be encountered crossing the aortic valve with the device during TAVI. However, it is not possible to know definitively whether there will be challenges until the delivery system is in the aortic root. At this point, the new valve may be irretrievable or require several backwards steps to remove, making efficient techniques to overcome the issue desired. Given that there has been demonstrated feasibility of standalone BAV being performed via TRA in certain circumstances,^7^ we explored the use of peripheral PTA balloons via TRA to facilitate aortic valve crossing. In Case 1, the inability to cross the valve related predominantly to severe tortuosity throughout the arterial tree and a subsequent lack of torque and device manipulation to negotiate the native valve. Strategies including the limited manipulation, changes in delivery system flex, minimal inflation of the deployment balloon, and a buddy wire were all unsuccessful. In Case 2, the issue largely related to the horizontal aorta and crossing was not possible despite pre-TAVI BAV with a suitably sized valvuloplasty balloon through the main access. Regardless of the underlying cause, this report demonstrates that balloon-assisted aortic valve crossing via TRA is feasible with both BE and SE TAVI platforms. Several plausible benefits and the limitations of the technique are described.

There is a temptation to use valvuloplasty balloons sized to the aortic annulus when balloon-assisted aortic valve crossing is being utilized. However, the issue commonly relates to the nosecone of the respective TAVI platform lodging peripherally in the commissure between the right- and non-coronary cusp or on the calcified leaflets themselves. Tracking around the greater curve of the aorta is commonly implicated, particularly in the case of a horizontal aorta. Only a small amount of displacement is usually necessary to move the nosecone into the valve orifice. Case 2 highlights this particularly well, with a small 8 mm PTA balloon that fits comfortably in a 6 Fr sheath sufficient to displace the delivery system and facilitate valve crossing.

In general, rather than ‘pushing harder’ and risking complications such as aortic or annular injury, a low threshold for this technique is proposed given its simplicity, low risk, and avoidance of a secondary femoral access. Our practice is to utilize BACTRA as part of a stepwise algorithm. Initially, general trajectory changes and other delivery system manipulations are attempted. For BE platforms, the cushion technique with a 1–2 mL inflation of the deployment balloon is then attempted. Thereafter, a buddy wire is trailed with the aforementioned strategies. If still unsuccessful, BACTRA can then be facilitated over this wire. By utilizing the technique at this time, there are minimal delays as the necessary second wire is already in the LV. Further, more complex manoeuvres, including device removal where feasible, gaining additional femoral access to facilitate a first or further BAV sized to the annulus, or loss of LV wire position to facilitate a snaring technique, can all be avoided if BACTRA is successful.

The radial approach for secondary access in TAVI is increasing in aid of lowering vascular complications.^8,9^ Nonetheless, limitations of the technique are similar to those experienced during TRA in invasive coronary angiography and percutaneous coronary intervention.^10,11^ Prior *trans*-radial interventions may have rendered the vessel occluded, calcified small radial arteries may not be of sufficient size to facilitate sheath insertion, and spasm may prevent advancement and manipulation of equipment, while radial loops or more proximal tortuosity may prevent use of TRA altogether. The BACTRA technique is rendered unsuitable in such instances. Nonetheless, where the pigtail catheter has been successfully advanced to the aortic root via TRA, it would be unusual in our experience not to be able to facilitate BACTRA. Spasm prophylaxis should be considered as with any TRA procedure, and therapeutic heparinization is standard practice during TAVI which will also minimize the risk of radial artery occlusion.^12^ Complications of TRA are infrequent and usually manageable with simple measures,^13^ noting we have not experienced any RA complications related to the BACTRA technique.

Where balloon-assisted valve crossing becomes necessary, our technique of passing a buddy balloon from the radial sheath maintains the benefit of secondary access via the RA. Avoiding another femoral puncture is likely to both lower vascular complication rates and maintain procedural efficiency given that this gentle escalation in crossing strategy can be facilitated on the buddy wire which had been inserted via the RA. In addition to facilitating the BACTRA technique, knowledge of the PTA balloon platforms and their sheath compatibility can aid in treating vascular complications, and these details are outlined in *Table 1*.

**Table 1:** Typical PTA balloon and sheath compatibilities, noting that the list is not exhaustive

Sheath size	Maximum PTA balloon diameter	Device	Maximum guidewire size
5 Fr	7 mm	Powerflex Pro (Cordis) Armada 35 (Abbott) Metacross OTW (Terumo) EverCross (Medtronic) Mustang (Boston Scientific)	0.035
	8 mm	Passeo-35 HP (Biotronic) Advance 35LP (Cook Medical)	
6 Fr	10 mm	Powerflex Pro EverCross Mustang Advance 35LP	0.035
	12 mm	Armada 25	
7 Fr	10 mm	Passeo-35 HP	0.035
	12 mm	Powerflex Pro Metacross OTW EverCross Mustang	
	14 mm	Armada 35 Atlas (Becton Dickinson)	
	15 mm	Valver (Balton)^a^	
8 Fr	12 mm	Passeo-35 HP	0.035
	18 mm	Atlas	0.035
	20 mm	Valver^a^	0.038

^a^The Valver balloon is specifically designed for balloon aortic valvuloplasty and is not a peripheral PTA balloon.

## Conclusions

The BACTRA technique to facilitate aortic valve crossing in TAVI using PTA balloons is safe and effective with both BE and SE TAVI systems. Such a strategy likely improves procedure efficiency and lowers vascular complication rates. Familiarity with PTA balloon and sheath compatibility is important, and specific nuances to optimize the chance of success are described in this case series.

## Supplementary Material

ytag536_Supplementary_Data

## Data Availability

The data underlying this article are available in the article and in its online Supplementary material.

## Lead author biography

Garry W. Hamilton is an interventional and structural heart disease cardiologist in Melbourne, Australia. He completed his fellowship at King’s College Hospital in London, UK.

## Notes

**Consent:** The authors confirm that written consent for submission and publication of this case report, including images and associated text, has been obtained from the patients in line with COPE guidance.
