# Supplementary material for: Balloon-assisted crossing via trans-radial access during transcatheter aortic valve implantation: a case series demonstrating the BACTRA technique
Source: Eur Heart J Case Rep. 2026 Aug 11;10(8):ytag536. doi: 10.1093/ehjcr/ytag536 (PMC13459947; doi:10.1093/ehjcr/ytag536)
Supplement: Supplementary file 1 [file ytag536_supplementary_data.zip › Supplemental video legends.docx]

**Videos**

**Video 1 – Case 1**

- **Title:** Infra-renal tortuosity in the aorta
- **Caption:** Left femoral access was chosen related to the infra-renal aortic angulation relative to the approach from the common iliac vessels. Despite this, difficulty was encountered exiting the sheath and advancing through the calcified and tortuous infra-renal aorta. The valve had to be loaded on the balloon in an unconventional position at the distal end of the sheath to facilitate this.

**Video 2 – Case 1**

- **Title:** Balloon expandable valve delivery to the aortic root
- **Caption:** Extreme iliac and aortic tortuosity made advancing the system challenging and significantly limited manipulation of the nosecone to adjust the trajectory due to a lack of transmission of movements.

**Video 3 – Case 1:**

- **Title:** Balloon assisted crossing of the aortic valve via TRA
- **Caption:** The 12 mm PTA balloon via TRA is inflated as forward traction is applied to advance the Sapien valve into the LVOT. No rapid pacing is necessary.

**Video 4 – Case 1:**

- **Title:** Final aortogram
- **Caption:** The valve was deployed successfully. Both invasive and echocardiographic haemodynamic were satisfactory with a low gradient and trivial paravalvular leak.

**Video 5 – Case 2:**

- **Title:** Balloon assisted crossing of the aortic valve via TRA
- **Caption:** The PTA balloon is manipulated onto the greater curve of the aorta ‘outside’ the Safari wire of the TAVI system by applying traction on the guidewire and PTA balloon catheter simultaneously. It is then inflated as the Navitor FlexNav system is advanced which displaces the nosecone into the valve orifice for smooth passage into the LVOT.

**Video 6 – Case 2:**

- **Title:** Final aortogram
- **Caption:** With the valve deployed, this final aortogram demonstrates the extremely horizontal aorta which influenced the trajectory and difficulty with valve crossing. Satisfactory valve deployment with no significant paravalvular leak or transvalvular gradient.
